# Intact microdissection of stellate ganglia in a Parkinson's disease model reveals aggregation of mutant human α‐synuclein in their cell bodies

**DOI:** 10.1113/EP092261

**Published:** 2025-02-21

**Authors:** Bonn Lee, Shiraz Ahmad, Charlotte E. Edling, Fiona E. N. LeBeau, Kamalan Jeevaratnam

**Affiliations:** ^1^ School of Veterinary Medicine, Faculty of Health and Medical Sciences University of Surrey Guildford UK; ^2^ Biosciences Institute, Faculty of Medical Sciences University of Newcastle, The Medical School Newcastle upon Tyne UK

**Keywords:** α‐synuclein, cardiac dysautonomia, Parkinson's disease, stellate ganglia, SUDPAR

## Abstract

Cardiac dysautonomia plays an important role in understanding Parkinson's disease (PD), with recent studies highlighting the presence of α‐synuclein in cardiac tissue. We hypothesise that sympathetic dysregulation observed in PD may involve pathological changes caused by α‐synuclein in stellate ganglia (SG). This study aimed to investigate α‐synucleinopathy in SG of the genetic PD murine animal model. Mice overexpressing Ala30Pro (A30P) mutant α‐synuclein were used. We here demonstrate a technique for meticulously dissecting SG. The collected SG from the transgenic mice were immunolabelled with neuronal markers, A30P human mutant α‐synuclein and anti‐α‐synuclein aggregates. A30P mutant α‐synuclein protein was expressed in the sympathetic neuronal (tyrosine hydroxylase (TH)‐positive) cell bodies. Approximately 27% of the TH‐positive cell bodies expressed the A30P mutant α‐synuclein protein. The mutant protein was densely localised at the cardiopulmonary pole of the SG. Additionally, we observed that the A30P mutant protein formed fibril aggregation in the SG. Our findings suggest that α‐synucleinopathy in the PD animal model can affect the sympathetic autonomic nervous system, providing insight for further research into targeting α‐synuclein pathology in the SG as a potential link between cardiac dysautonomia and PD.

## Abstract (highlights)

- **What is the central question of this study?**
  Does sympathetic dysregulation observed in Parkinson's disease (PD) involve pathological changes caused by α‐synuclein in stellate ganglia?
- **What is the main finding and its importance?**
  α‐Synucleinopathy in a PD animal model can affect the sympathetic autonomic nervous system, providing insight for further research into targeting α‐synuclein pathology in the stellate ganglia as a potential link between cardiac dysautonomia and PD.

## 1 INTRODUCTION

Parkinson's disease (PD) is now widely accepted to manifest with a constellation of systemic neuropathology (Chaudhuri et al., 2006; Jost, 2003; Schapira et al., 2017) in addition to central nervous system (CNS) changes. Importantly, cardiovascular diseases have been increasingly recognised as a key non‐motor feature of PD (Lee et al., 2023; Scorza et al., 2018). Arrhythmia and sympathetic denervation are considered as cardiac features of PD (Jain & Goldstein, 2012; Nishida et al., 2017). Cardiac autonomic dysregulation in PD is thought to play a pivotal role in understanding PD (Lee et al., 2023). Earlier research demonstrated that PD patients present with a reduction in the tyrosine hydroxylase (TH)‐positive sympathetic neurons in the heart (Amino et al., 2005; Takahashi et al., 2015), and recent findings have established PD‐associated pathology, such as Lewy bodies (LBs), in the heart (Javanshiri et al., 2022).

LBs, the pathological hallmark of PD, were first identified in the dorsal vagal nucleus and nucleus basalis, and it is now recognised that LBs can present in any organs innervated by the autonomic nervous system (Borghammer et al., 2021). α‐Synuclein is the major constituent of LBs, and missense mutations of the gene are known to aggravate protein misfolding to form LB (Mehra et al., 2019; Wakabayashi et al., 2013).

Ala30Pro (A30P) point mutation in the α‐synuclein (*SNCA*) gene is already known as a genetic risk factor of PD, which contributes to the protein misfolding (Kruger et al., 1998; Pankratz et al., 2009). Mice overexpressing the human A30P mutant α‐synuclein are well established as a PD animal model (Kahle et al., 2000). The mice recapitulate α‐synuclein deposition in brain with subsequent changes in animal behaviour tests (Ekmark‐Lewen et al., 2018). Additionally, the mouse model develops α‐synuclein aggregates in mesenteric plexus with dysregulations of intestinal movements, which implies that the mice could also reflect the peripheral neuronal features of PD (Gries et al., 2021).

Stellate ganglia (SG) provide autonomic input to the heart (Rajendran et al., 2019). Inflammation of the SG has been linked to dysregulation of cardiac sympathetic outflow, which might result in life‐threatening disease such as sudden cardiac death (Duffy et al., 2021; Rizzo et al., 2014). In disease animal model studies, molecular remodelling of SG indicated an alteration of the heart rhythm or heart failure (Ajijola et al., 2015; Sharma et al., 2023). We aimed to investigate whether the α‐synuclein A30P mutation impacts the stellate ganglia at the protein level and the formation of its pathological aggregates, which potentially brings molecular remodelling of SG.

In this study, we aimed to investigate the stellate ganglia using the A30P human mutant α‐synuclein mouse because of its known α‐synuclein deposition in both CNS and peripheral nervous system. We here demonstrate a technique for meticulously dissecting the SG for further molecular analysis. The collected SG were immunolabelled with neuronal markers to examine the neurochemical properties, and we also investigated the distribution of the A30P human mutant α‐synuclein protein in SG.

## 2 METHODS

### 2.1 Ethical approval

All animal‐related research procedures in this study were done in accordance with the UK Animals (Scientific Procedures) Act 1986 and European Union directive 2010/63EU. Ethics approval was received from the Animal Welfare Ethical Review Body (AWERB) of the University of Surrey (NASPA‐1819‐25 Amend 1) for investigating the C57BL/6 wild‐type (WT) mice and from the AWERB Newcastle University for the A30P transgenic mice. All investigators understand the ethical principles under which the journal operates and certify that the present study complies with their animal ethics checklist.

#### 2.1.1 Animal management

Transgenic mice of C57BL/6 background expressing human mutant A30P α‐synuclein ([A30P]αSYN) were generated under the control of the CNS specific promotor Thy‐1 (Kahle et al., 2000). The A30P mice homozygous breeding pairs were originally supplied by Dr P. Kahle (University of Tubingen) and were housed in the animal facility at Newcastle University. The mice were kept in an enriched environment (cage toys) on a 12‐h light–dark cycle with lights on at 07.00 h and with access to food and water ad libitum. Five male and four female 4‐month‐old transgenic mice were used for the study. The transgenic mice were obtained from three different litter groups. Age‐ and sex‐matched C57BL/6 WT controls (five male and four female) were supplied from Charles River Ltd (Charles River UK Ltd, Margate, UK). The WT mice were maintained in the University of Surrey Biological Resource Facility under controlled conditions with ambient temperature 23 ± 2°C, 12‐h light–dark cycle with access to food and water ad libitum. All the procedures were performed conforming to the guidelines from Animals (Scientific Procedures) Act 1986 (UK) and NIH *Guide for the Care and Use of Laboratory Animals*.

### 2.2 Dissection of tissues

Mice were euthanised by cervical dislocation, and the death of the animals was confirmed by permanent cessation of the circulation (as per the Animals (Scientific Procedures) Act 1986 (UK)), then the thoracic cavity was opened for collecting the tissues. After removing adipose tissues in superior mediastinum, SG were located (Supporting information Video S1). SG and surrounding tissue were completely removed using micro scissors (cat. no. 15906: World Precision Instrument, Sarasota, FL, USA), and the SG were then separated from the tissue aggregate using tweezers (cat. no. 14101, World Precision Instrument). The tissues were fixed in 4% paraformaldehyde and transferred to phosphate‐buffered saline (PBS) with 30% sucrose for 24 h for dehydration. The tissues were embedded with cryopreservation compound (cat. no. 4585: Sigen Scientific, Gardena, CA, USA) and snap‐frozen in liquid nitrogen. The tissues were sectioned to a thickness of 6 µm by coronal plane using a Hydrax‐C Cryotome (43961, Zeiss, Oberkochen, Germany) and placed on the slide glasses (Superfrost Plus Adhesion Microscope Slides, cat. no. J1800AMNZ, New Erie Scientific, Portsmouth, NH, USA). For quality control among the tissues, slides obtained from 24 µm before and after the largest cross‐sectional area were used in the experiment. Thus, six to eight microscope slides were obtained from one stellate ganglion.

### 2.3 Haematoxylin and eosin staining

The sections were washed with PBS to remove antifreeze, then rehydrated in descending graded ethanol solutions. The tissues were stained with Harris haematoxylin solution (cat. no. HHS16, Sigma‐Aldrich, St Louis, MO, USA) according to the manufacturer's protocol, then dipped into eosin solution (cat. no. RBC‐0100‐00A, CellPath Ltd, Newtown (Powys), UK) for counter‐staining.

### 2.4 Immunofluorescence

The tissues were boiled in acidic (pH 6.0) citrate buffer (10 mM, tri‐sodium citrate dihydrate, cat. no. 27833.260, VWR Chemicals, Leuven, Belgium) for antigen retrieval. Antigen retrieval was not performed for the conformation‐specific antibody (anti‐α‐synuclein aggregates). Tissues were blocked with 2.5% normal bovine serum in PBS for 1 h, then incubated with primary antibodies. All the antibodies were diluted in 1% normal bovine serum in PBS, and the dilution rates were the following: α‐synuclein human monoclonal antibody‐15G7 (1:125 dilution, overnight at −4°C, cat. no. ALX‐804‐258, Enzo Life Science, Farmingdale, NY, USA), tyrosine hydroxylase (TH) polyclonal antibody (1:250, cat. no. PA5‐18372, Thermo Fisher Scientific, Waltham, MA, USA), vasoactive intestinal peptide (VIP) polyclonal antibody (1:250, cat. no. PA1‐85958, Thermo Fisher Scientific), neuronal nitric oxide synthase (nNOS) polyclonal antibody (1:250, overnight, PA1‐033, Thermo Fisher Scientific), anti‐α‐synuclein aggregate [MJFR‐14‐6‐4‐2] (1:5000, 2 h, cat. no. ab209538, Abcam, Cambridge, UK), and anti‐Thy‐1 monoclonal (1:500, overnight, cat. no. ab307736, Abcam). For fluorescence labelling, slides were incubated with secondary antibodies for 1 h. Slices were incubated with PBS containing 6‐diamidino‐2‐phenylindole dihydrochloride (DAPI; cat. no. MBD0015‐1ML, Sigma‐Aldrich). The secondary antibodies details are as follow: Alexa Fluor 488 donkey anti‐rat IgG secondary antibody (1:2000, cat. no. A‐21208, Thermo Fisher Scientific), Alexa Fluor 568 donkey anti‐rabbit IgG secondary antibody (1:2000, cat. no. A10042, Thermo Fisher Scientific) and Alexa Fluor 488 donkey anti‐goat IgG secondary antibody (1:2000, cat. no. A11055, Thermo Fisher Scientific). Slides were counter‐stained with 1% Sudan black solution to reduce the autofluorescence and then covered with coverslips using Mowiol mounting medium (cat. no. 81381, Sigma‐Aldrich). Negative controls were prepared each time by omitting primary antibodies to assess autofluorescence from tissue background or non‐specific binding.

#### 2.4.1 Quantification of A30PαSYN positivity on the stellate ganglia tissue slides

The microscopic images of the stellate ganglia were captured using the Eclipse Ci Microscope System (Nikon Instruments, Tokyo, Japan) at ×100 magnification. SG tissues were positioned entirely within one microscope field at ×100 magnification, then images were captured with cardiovascular pole at the centre of the microscope field of view as the reference point. The fluorescence images were split by RGB channel, and each image was converted to binary colour using NIH ImageJ software (see Supplementary Figure 2 and 3). The number of [A30P]αSYN‐positive cell bodies and TH‐positive cell bodies (total cell bodies) were counted using the cell counter function in ImageJ. There were differences in total cell body counts depending on the position of each slide (minimum 100 to maximum 306 counts). To adjust for the differences and allow data comparison across the individual samples, the number of [A30P]αSYN‐positive cells was normalised by that of total cell bodies. The normalised cell body count was expressed as whole numbers. Five male and four female SG slides were investigated. Each slide from the independent animal was counted as *n* of 1.

### 2.5 Statistical methods and data handling

The quantified data were compared between WT and the transgenic mice, and the statistical significance was calculated using GraphPad Prism 9.1.0. (GraphPad Software, Boston, MA, USA). The mean cell counts between wild‐type and genotype mice were compared using Student's *t*‐test. To compare the mean cell counts between males and females, the Shapiro–Wilk normality test was applied due to the small sample size (*n* = 9) to determine if the data followed a normal distribution. After confirming normality, comparisons were made using Student's *t*‐test. For the statistical analysis, no outliers were identified and removed from the curated dataset. All quantitative data were included for statistics.

## 3 RESULTS

### 3.1 Isolation and verification of murine stellate ganglia

**FIGURE 1:**
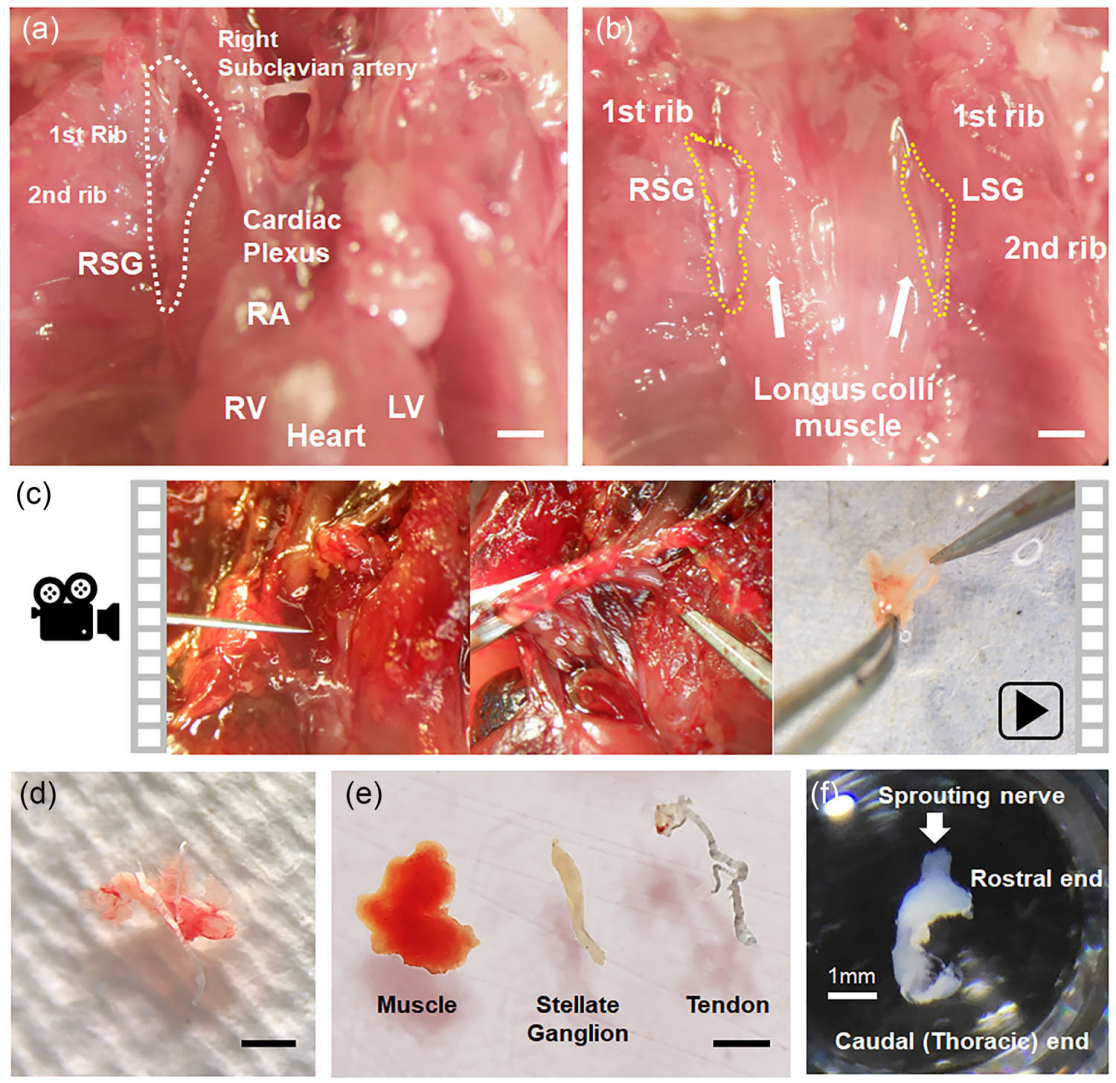
Anatomical identification of stellate ganglion. (a) Anatomical positions of stellate ganglion linked to heart. RSG (white‐line) is connected with heart via cardiac plexus, RA, RV and LA. Scale bar 2 mm. (b) Heart removed to expose right (RSG) and left (LSG) stellate ganglia marked with yellow line. Scale bar 2 mm. (c) A time‐lapse demonstration guide for the dissection; for full‐version video, see Supporting information Video S1. (d) A lump of tissue surrounding stellate ganglion. Scale bar 2 mm. (e) Right stellate ganglion after trimming; comparison of muscle, stellate ganglia and tendon; tissues were collected from the adjacent area. Scale bar 2 mm. (f) Right stellate ganglion with stereomicroscopy imaging. Scale bar 1 mm. LA, left atrium; RA, right atrium; RSG, right stellate ganglia; RV, right ventricle.

SG were harvested from 4‐month‐old [A30P]αSYN mice to evaluate neuronal protein expression in autonomic ganglia (Figure 1). SG are located where the costal groove of rib bones and longus colli muscle form a ridge (Hedger & Webber, 1976; Scherschel et al., 2020). The thoracic cavity was opened, and the thymus and superior mediastinal adipose were removed, then the right SG was identified as fusiform‐shaped and placed horizontally to the longus colli muscle. We observed that connective tissue and minute neuronal tissue (rami communicantes) were branched from the right stellate ganglion (Figure 1a, white dashed line), which was previously described as the cardiac pole (Sharma et al., 2023). After removing the heart, sympathetic trunks containing SG were identified parallel and adjacent to the longus colli muscle (Figure 1b). However, for inexperienced researchers, SG may not be easily distinguishable by their colour or texture from the connective tissues, which might lead to damage of the tissue during blunt separation for locating SG. Previously, Scherschel et al. (2020) demonstrated locating and dissecting SG (Scherschel et al., 2020). Here, SG were harvested with surrounding tissues using micro scissors without performing blunt separation on the carcass to minimise damaging the tissue (Figure 1c and Supporting information Video S1). SG was then trimmed by removing blood clots and debris (Figure 1d). From the tissue aggregate, the SG, with a fusiform shape and sprouting nerve branches, was then distinguished (Figure 1e). The stellate ganglion is 3 mm in length with a sprouting nerve branch, which Rajendran et al. (2019) reported is linked to the heart and provides sympathetic regulation (Rajendran et al., 2019) (Figure 1f).

### 3.2 Histological observation of murine stellate ganglia

**FIGURE 2:**
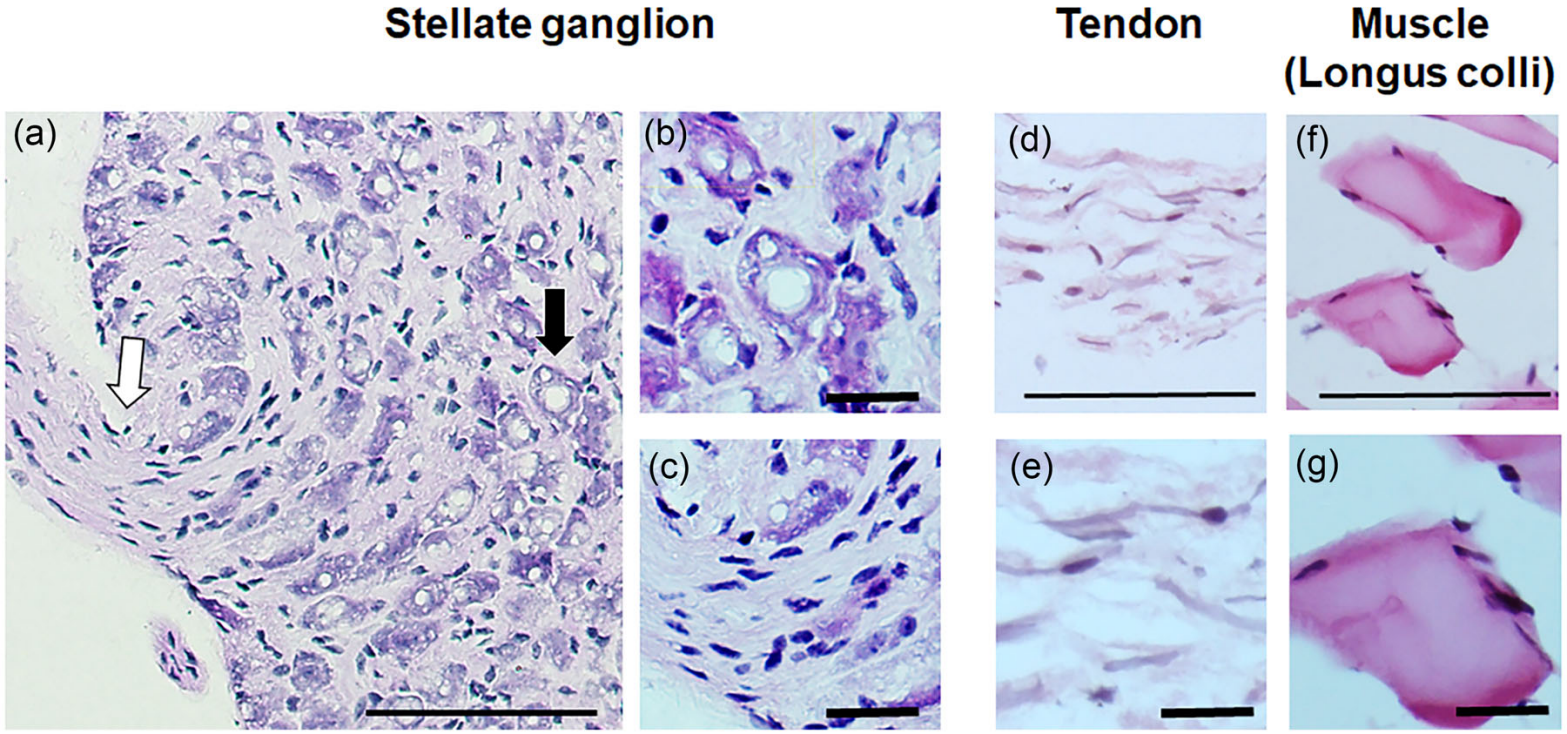
Histological identification of stellate ganglion. (a) Haematoxylin and eosin staining for stellate ganglion. Neuronal cell body, black arrow; nerve branch projecting to heart, white arrow. (b) Neuronal cell body (magnification of black arrow area in (a)). (c) Nerve branch projecting to heart (magnification of white arrow area in (a)). (d) Tendon. (e) Tendon at a higher magnification. (f) Longus colli muscle. (g) Muscle at a higher magnification. Scale bar in (a, d, f), 100 µm scale bar in (b, c, e, g), 20 µm. Tissues were collected from 4‐month‐old mice.

The tissue aggregate containing SG was histologically identified with haematoxylin and eosin staining (Figure 2). Histological verification for the tissue is highly recommended because SG may not be distinguishable from the surrounding tissues by its appearance. In the SG, neuronal cell bodies (soma; Figure 2a, black arrow; Figure 2b) and interstitial cells (Figure 2a white arrow; Figure 2c) are clustered, while the other tissues do not have the structure of neuronal cells (Figure 2d,g).

### 3.3 Sympathetic adrenergic marker protein expression in neuronal cell body in stellate ganglia

**FIGURE 3:**
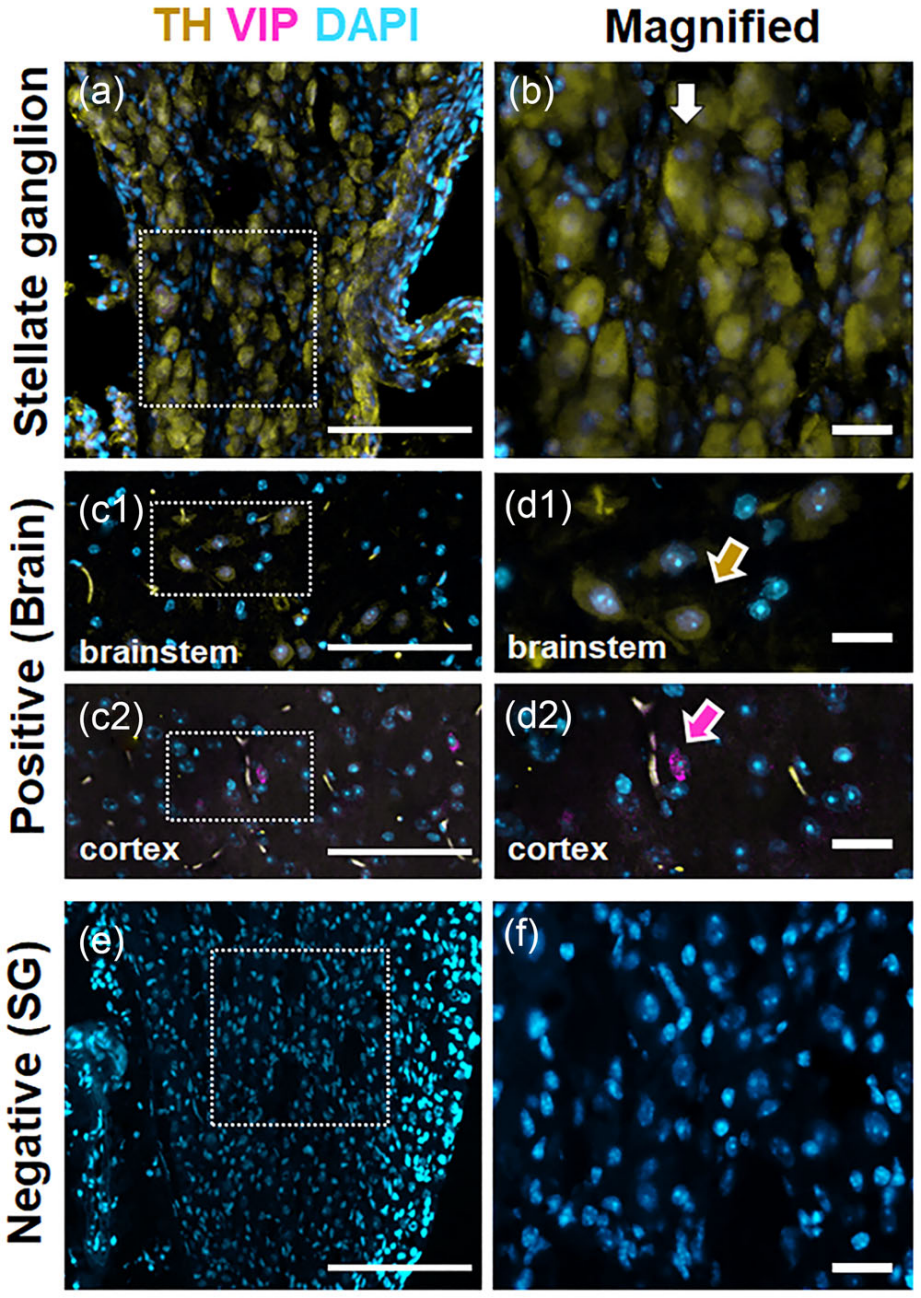
Sympathetic and parasympathetic marker expression in stellate ganglion. Sympathetic marker TH and parasympathetic marker VIP were co‐stained in stellate ganglion. Nuclei were stained with DAPI. (a) Stellate ganglion. TH was detected in neuronal cell bodies. (b) Higher magnification of a neuronal cell body (white arrow). (c1) Positive control, cerebral cortex. (c2) Positive control, brainstem. (d1) Higher magnification of c1, TH‐positive cell body (yellow arrow). (d2) Higher magnification of c2, VIP‐positive cells (magenta arrow). (e) Negative control in stellate ganglion. (f) Higher magnification of (e). Scale bar 100 µm in (a, c1, c2, e); scale bar 20 µm in (b, d1, d2, f). DAPI, 4′,6′‐diamidino‐2‐phenylindole; TH, tyrosine hydroxylase; VIP, vasoactive intestinal peptide.

To investigate the autonomic characteristics of the nerve cells in SG, the sympathetic adrenergic marker TH and the parasympathetic marker vasoactive intestinal peptide (VIP) were stained in SG (Cuthbertson et al., 2003). The immunolabelling showed TH in the nerve cell bodies (Figure 3b, white arrow), while VIP was not present in SG. We also investigated a parasympathetic marker (Navickaite et al., 2021), neuronal nitric oxide synthase (nNOS). nNOS was not expressed in the SG (Supporing information Figure S1). To ensure proper staining, we included brain tissue as an external positive control and no primary antibody control as a negative control. TH (Figure 3d, yellow arrow) and VIP (Figure 3d, magenta arrow) proteins were expressed in brain, while no antibody signal was detected on the negative slide (Figure 3e–f). Conclusively, we observed that the dissected tissues expressed sympathetic adrenergic markers in neuronal cell bodies and did not express parasympathetic neuronal markers. Since postganglionic sympathetic neurons are known not to have multiple neurochemical phenotypes simultaneously, we confirmed that the neuronal cell bodies in our SG samples are mainly sympathetic (Clyburn et al., 2022).

### 3.4 Mutant α‐synuclein protein expression in the sympathetic adrenergic cells

**FIGURE 4:**
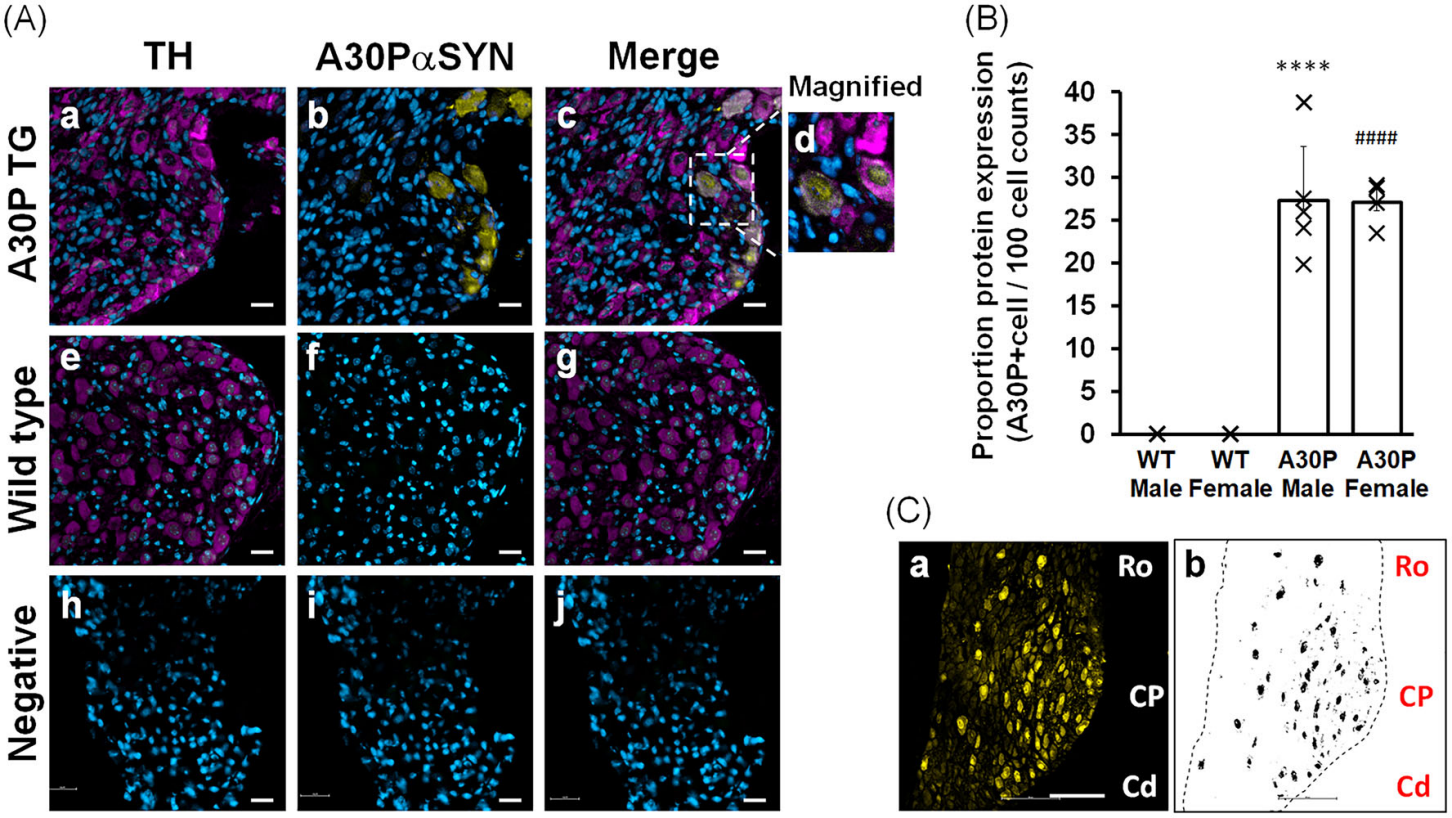
A30P mutant α‐synuclein protein expression in stellate ganglia of the Parkinson's disease animal model. (A) Representative immunofluorescence images of A30P mutant α‐synuclein protein expression in stellate ganglia. Anti‐human α‐synuclein ([A30P]aSYN) and anti‐TH were immunolabelled in stellate ganglia. Scale bar 20 µm. Nuclei were stained with 4′,6′‐diamidino‐2‐phenylindole. (a–d) A30P mutant; (e–g) wild‐type mice; (h–j) negative control, A30P mutant mouse tissue. (B) Quantification of [A30P]aSYN‐positive cells in stellate ganglia. [A30P]aSYN‐positive cell bodies were counted and normalised by the total number of cell bodies in each microscopic field. 27.12 cells per 100 cell bodies were [A30P]aSYN‐positive in the stellate ganglia of the transgenic mouse (A30P), while all the cells were [A30P]aSYN‐negative in the WT mice. Statistical significance between WT and A30P, *t*_12 _= 11.06, ***P = 0.00012 WT versus A30P (WT male vs. A30P male, *t*_8 _= 8.76, ****P < 0.0001; WT female vs. A30P female, *t*_6 _= 16.97, ^####^ *P < 0.0001*). Crosses represents the individual data. Five male mice (*n* = 5) and four female mice (*n* = 4) were investigated in each genotype; error bar represents the standard deviation. (C) Representative images illustrating the [A30P]aSYN protein distribution in the stellate ganglia. (a) The [A30P]aSYN‐positive cells were located by yellow fluorescence. (b) The binary converted image of (a). Black dots reveal the mutant protein positive‐cells; the tissue edge is contoured with the dashed line. Scale bar, 100 µm. Cd, caudal; CP, cardiopulmonary pole; TH, tyrosine hydroxylase; Ro, rostral; WT, wild‐type.

To confirm the presence of [A30P]αSYN in SG, we immunolabelled the tissue with human anti‐α‐synuclein antibody. The [A30P]αSYN mouse is generated to express the human A30P point mutant α‐synuclein (Kahle et al., 2000). The anti‐human α‐synuclein antibody was used to selectively detect [A30P]αSYN protein. Anti‐TH antibody was co‐stained to locate the sympathetic adrenergic cells. We observed that anti‐[A30P]αSYN and anti‐TH were both detected in the neuronal cell bodies (Figure 4Aa–d). In contrast, WT tissue did not express the [A30P]αSYN in the SG tissue (Figure 4Ae–g). We found that mutant [A30P]αSYN protein was expressed in the adrenergic neuronal cell bodies of SG in the A30P mutant mice. However, not all the cell bodies expressed the mutant [A30P]αSYN. Then, we investigated whether the protein was expressed in non‐neuronal cell bodies; the images were converted to binary colour to enable distinguishing minute expression, and we confirmed that [A30P]αSYN protein was expressed solely in the soma (Supporting information Figure S2). From the binary processed images (Supporting information Figure S2), relative protein expression of the [A30P]αSYN was quantified. The number of A30PαSYN‐positive cells in each captured image were counted and normalised against the total number of neuronal cell bodies (Figure 4B). Both male and female A30P mice expressed [A30P]αSYN protein in stellate ganglia (no significant difference in the cell counts number by sex, *t*_7 _= 1.159, *P *= 0.495). We quantified the proportion of [A30P]αSYN‐positive neurons against the total TH‐positive neurons in the SG. In the [A30P]αSYN male mice, the cell count was 27 ± 6 per 100 cells (mean ± standard deviation, *n* = 5, *P* < 0.0001 compared to WT males). In the transgenic female mice, the cell count was 27 ± 2 per 100 cells (mean ± standard deviation cell bodies, *n* = 4, *P* < 0.0001 compared to wild‐type females). The cell counts in the WT mice were 0. There was no significant difference in the [A30P]αSYN cell count number between male and female (*t*_5.252_ = 0.051, *P* = 0.9612). Interestingly, the mutant proteins were abundantly distributed at the cardiopulmonary pole (Figure 4C and Supporting information Figure S3).

### 3.5 Investigation of the transgene overexpression promoter Thy‐1 activity in stellate ganglia

**FIGURE 5:**
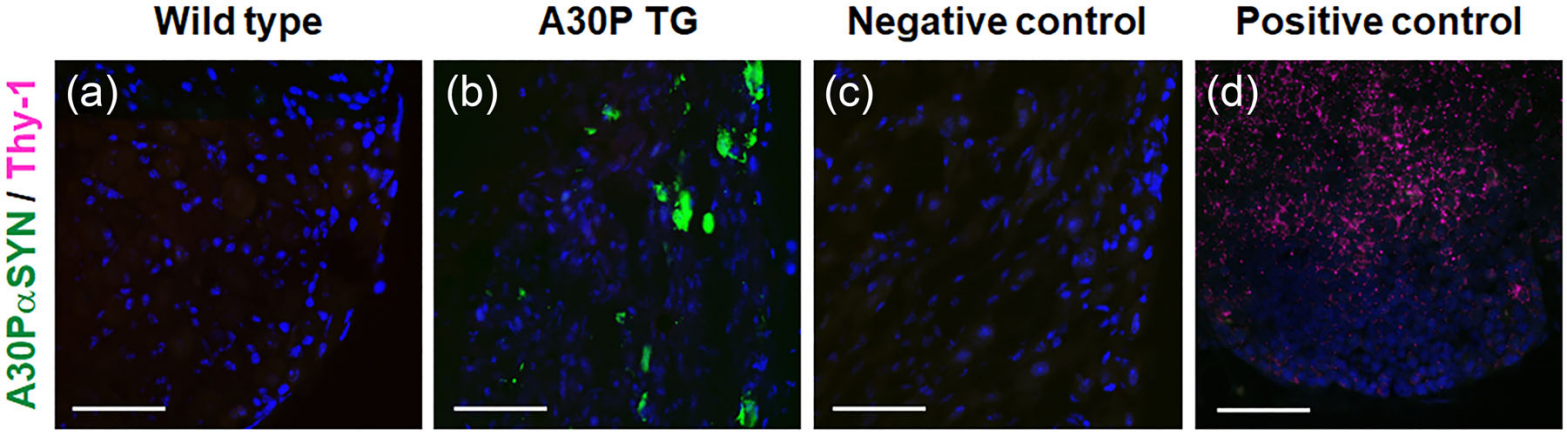
The transgene promoter Thy‐1 was not detected in stellate ganglia in the A30P transgenic mice. The gene overexpression promoter Thy‐1 cell surface antigen (mouse) was immunolabelled by using anti‐THY1 and anti‐[A30P]aSYN. (a) wild‐type mice; (b) A30P transgenic mouse; (c) negative control; (d) positive control. Thy‐1 protein was not detected in the stellate ganglia; wild‐type thymus tissue was used as a primary antibody positive control for validation of the primary antibody reactivity, and A30P transgenic tissue was used as a negative control. Scale bar 50 µm.

After we observed the [A30P]αSYN protein expression, we investigated the activity of the transgene overexpressing promoter in SG tissue (Figure 5). The A30P transgenic line was generated by employing human Thy‐1 promoter for tissue‐specific gene delivery to neuronal cells (Kahle et al., 2000; Vidal et al., 1990). Transgenic mice with the Thy‐1 promoter have been known to express a Thy‐1 hybrid gene sequence and protein in brain (Vidal et al., 1990). Thymus tissue was stained with anti‐Thy‐1 for technical verification as the primary antibody positive control (Figure 5d) (Kollias et al., 1987; Vidal et al., 1990). Thy‐1 protein was not detected at the stellate ganglia of the transgenic mice (Figure 5b). This implies that the transgene promoter was inactive in the stellate ganglia.

### 3.6 A30P mutant α‐synuclein forms aggregates in stellate ganglia

**FIGURE 6:**
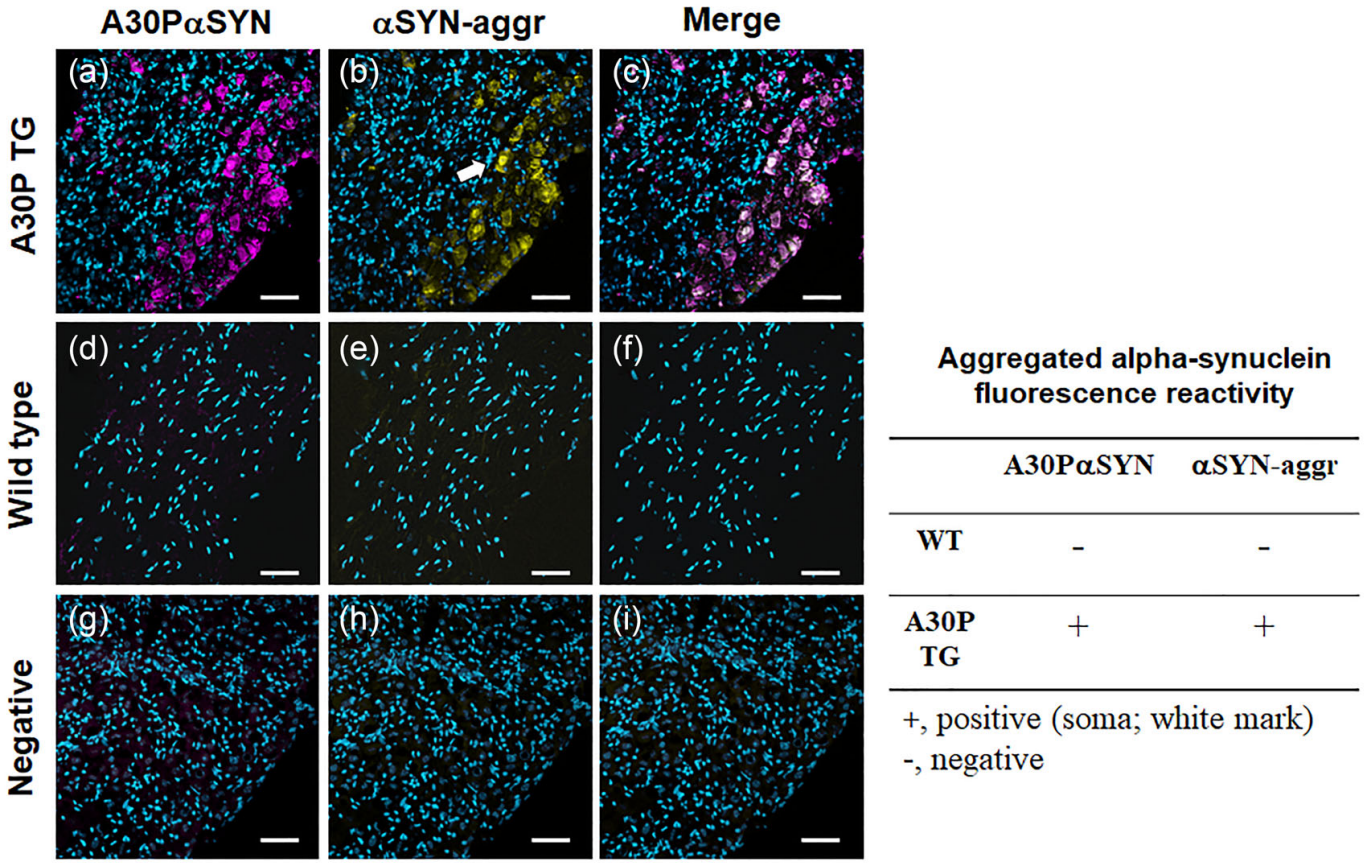
The A30P mutant α‐synuclein forms aggregates in mouse stellate ganglia. Anti‐human α‐synuclein ([A30P]aSYN) and anti‐α‐synuclein aggregates (aSYN‐Aggr; conformation‐specific antibody) were co‐localised in stellate ganglia. White arrow, positive fluorescence signal detected at soma. Scale bar 50 µm. Nuclei were stained with 4′,6′‐diamidino‐2‐phenylindole. (a–c) A30P mutant; (d–f) wild‐type mice; (g–i) negative control, A30P mutant tissue.

We investigated whether the A30P mutant protein formed α‐synuclein aggregates in SG. A30P mutant α‐synuclein has a higher propensity to form fibrillar aggregate than naïve α‐synuclein due to its different protein conformational dynamics (Li et al., 2001; Narhi et al., 1999). The conformational‐specific antibody against the aggregate form of α‐synuclein was co‐localised with anti‐[A30P]αSYN in SG tissues (Figure 6). Interestingly, the aggregate site‐specific antibodies were detected at the [A30P]αSYN‐positive cell bodies (Figure 6c). The aggregated α‐synuclein presented in the soma (Figure 6b, white arrow) in the A30P mutant. In contrast, the aggregate form of α‐synuclein was not detected in the WT SG. These findings suggest that the A30P mutant protein forms fibrillar aggregation in SG.

## 4 DISCUSSION

Accumulating evidence supports cardiac dysautonomia in PD patients as playing an important part in understanding Parkinsonism (Lee et al., 2023; Li et al., 2021; Scorza et al., 2018). As SG innervate the heart and regulate sympathetic input, investigating SG could play a pivotal role in understanding neurocardiac dysregulation in PD (Barrett et al., 2022; Rajendran et al., 2019). Surgical approaches to SG are considered to correct cardiac dysautonomia (Elliott et al., 2021; Lee et al., 2022). Although several mouse disease models have been generated for investigating cardiac dysautonomia (Blackwell et al., 2022), the importance of the SG has been neglected due to the shortage of information and the technical difficulty of the microdissection. We have here described a method to accurately isolate the SG from the [A30P]αSYN transgenic mouse, which is an established PD animal model. Our intact microdissection enables the collecting of SG tissue with minimal damage to the tissue. We expect that in disease animal model research this information will be useful for biological analysis requiring SG tissue in an intact condition and shape, such as electrophysiological investigation or high‐throughput RNA sequencing. In addition, we demonstrated that the [A30P]αSYN mutant protein can be expressed partially in the TH‐positive cell bodies in the SG, and the mutant α‐synuclein formed fibril aggregates in the PD animal model.

On reflecting on the potential research value of the [A30P]αSYN mouse, we think that the mouse will be of value as a useful research tool for investigating dysautonomia. First, the deposition of α‐synuclein aggregates in adrenergic neuronal cells in the SG may be associated with potential consequences mediated by α‐synuclein pathology within the sympathetic autonomic nervous system. Seeding α‐synuclein in the SG by injection resulted in autonomic dysregulation across multiple organs, leading to hypotension and constipation (Wang et al., 2020). In PD research, cardiac dysautonomia is still a debated topic; it could be secondary to CNS dysfunction, or autonomic degeneration may be the primary reason (Goncalves et al., 2022). However, a body of literature indicates that LBs play a role in cardiac dysautonomia in PD patients (Goncalves et al., 2022). In cardiac dysautonomia patients, ganglionitis in stellate ganglia has been linked to arrythmia and sudden cardiac death (Duffy et al., 2021; Rizzo et al., 2014). Interestingly, Rizzo et al. (2014) observed ganglionitis in their arrhythmia patients, albeit they traced the remnants of a possible viral infection to explore the cause of the ganglionitis and the reason for the infection was unresolved. Aggregation of α‐synuclein is thought to be a fundamental step in PD‐mediated neurodegeneration; the α‐synuclein aggregates are predicted to promote complicated interplay of post‐translational modifications to form LB (Mahul‐Mellier et al., 2020). α‐Synucleinopathy per se can trigger neuroinflammation and cellular stress responses (Klegeris et al., 2008; Virdi et al., 2022). In this regard, the α‐synuclein aggregates developed in the SG in the PD mouse model that presents α‐synucleinopathy in SG can form an important tool for further research to study cardiac dysautonomia in PD.

Second, we demonstrated that, in the [A30P]αSYN mice, α‐synuclein aggregates developed in the sympathetic autonomic system. This is the first report of α‐synucleinopathy in murine sympathetic autonomic nerves in a genetic PD animal model. According to Braak's theory, the dorsal motor nucleus of vagus (DMV) is known as the primary target of LBs (Braak et al., 2003). Therefore, PD animal model studies have focused on recapitulating parasympathetic features in PD, which are regulated by DMV (Gries et al., 2021; Kim et al., 2019). Few studies have focused on the sympathetic system in PD animal model studies; however, the animal model presented α‐synuclein pathology only in the CNS region (Fleming et al., 2013). On the other hand, in human post‐mortem studies, α‐synuclein pathology in SG has been importantly addressed. Miki et al. (2009) observed α‐synuclein‐positive LB deposition in SG in a 35‐year‐old man who died incidentally without Parkinsonism or dysautonomia (Miki et al., 2009). In addition, Beach et al. (2010) report α‐synucleinopathy in human cervical ganglia in PD subjects (Beach et al., 2010). Earlier studies exploring the SG indicated the sympathetic autonomic alteration can be associated with α‐synucleinopathy in PD. Admittedly, the current study did not provide the mechanistic link between the sympathetic autonomic function and α‐synuclein pathology of SG due to lack of a functional study in the animal model. Therefore, future research should focus on the functional impact of α‐synuclein aggregation in SG in the PD animal model, which we expect will provide insights into sympathetic dysautonomia in PD.

Third, the presence of α‐synuclein in SG raises the question whether SG play a role in α‐synuclein propagation. In this study, the A30P mutant proteins were abundantly located in the cardiopulmonary pole of SG, and only some of the cell bodies (27.1 per 100 cells) presented the mutant protein aggregation. We observed that the transgene promoter THY‐1 protein was not expressed in the SG, which casts doubt on the origin of the mutant protein. Recent studies report that SG themselves could be the origin of α‐synucleinopathy, independently of the cranial pathology (Miki et al., 2009; Sumikura et al., 2015). Sumikura et al. (2015) suggested that α‐synuclein‐positive LB might be retrogradely spread from sympathetic preganglionic nerves to the dorsal root of the spinal cord (Sumikura et al., 2015). In their studies, the presence of LB in the SG was correlated with that in the thoracic spinal cord, but some subjects presented LB in the SG without presenting it in the brain and spinal cord (Sumikura et al., 2015). Additionally, Miki et al. (2009) support that α‐synucleinopathy may develop in SG independently from other parts of body since α‐synuclein‐positive LBs were observed in SG and the heart, but not detected in any other visceral organs of the subject (Miki et al., 2009). Considering the function and anatomical proximity of SG to heart, it is plausible that the α‐synuclein pathology can be transmissible to the heart, or from the heart. Cardiac‐presenting α‐synucleinopathy can support the transmission of α‐synuclein via the SG and has been reported in human subjects with and without Parkinsonism (Iwanaga et al., 1999; Javanshiri et al., 2022; Navarro‐Otano et al., 2013). Wang et al. (2020) demonstrated this transmission of α‐synuclein from the SG to the heart by inoculation of exogenous α‐synuclein aggregates to the murine SG (Wang et al., 2020). Since our PD animal model involved α‐synuclein aggregation driven by the overexpression of the protein within neurons, it is necessary to verify the protein transmission process in further studies.

In conclusion, our observation that the [A30P]αSYN mice present α‐synuclein aggregates in the SG highlights the potential of this animal model for use in further research. While this study focused on immunohistochemical analyses of SG tissue, we anticipate that further investigations into the molecular alterations are required to provide biological consequences of α‐synuclein aggregates in the SG of the animal disease model.

## AUTHOR CONTRIBUTIONS

All authors had full access to all data in the study and take responsibility for the integrity of the data and the accuracy of the data analysis. Conceptualization: Bonn Lee, Kamalan Jeevaratnam. Data curation: Bonn Lee, Shiraz Ahmad. Investigation: Bonn Lee. Formal analysis: Bonn Lee. Resources: Fiona E. N. LeBeau. Writing original draft: Bonn Lee. Writing review and editing: Shiraz Ahmad, Charlotte E. Edling, Fiona E. N. LeBeau. Visualization: Bonn Lee, Shiraz Ahmad. Project administration: Charlotte E. Edling. Funding acquisition: Kamalan Jeevaratnam. Supervision: Kamalan Jeevaratnam. All authors have read and approved the final version of this manuscript and agree to be accountable for all aspects of the work in ensuring that questions related to the accuracy or integrity of any part of the work are appropriately investigated and resolved. All persons designated as authors qualify for authorship, and all those who qualify for authorship are listed.

## CONFLICT OF INTEREST

The authors declare that they have no conflict of interest.

## Supporting information



Supplementary Figure 1. Immunolabelling of sympathetic and parasympathetic markers in murine stellate ganglia.
Supplementary Figure 2. Investigation of TH and hαSYN overlapping area in stellate ganglia in [A30PαSYN] mice.
Supplementary Figure 3. The mutant protein distribution in the cardiopulmonary pole of stellate ganglia.

Microdissection demonstration of the murine stellate ganglion

## Data Availability

All data generated or analysed during this study are included in this article and its supporting information.

## References

[eph13768-bib-0001] Ajijola, O. A. , Yagishita, D. , Reddy, N. K. , Yamakawa, K. , Vaseghi, M. , Downs, A. M. , Hoover, D. B. , Ardell, J. L. , & Shivkumar, K. (2015). Remodeling of stellate ganglion neurons after spatially targeted myocardial infarction: neuropeptide and morphologic changes. Heart Rhythm, 12(5), 1027–1035. 25640636 10.1016/j.hrthm.2015.01.045 PMC4411181

[eph13768-bib-0002] Amino, T. , Orimo, S. , Itoh, Y. , Takahashi, A. , Uchihara, T. , & Mizusawa, H. (2005). Profound cardiac sympathetic denervation occurs in Parkinson disease. Brain Pathology, 15(1), 29–34. 15779234 10.1111/j.1750-3639.2005.tb00097.x PMC8095848

[eph13768-bib-0003] Barrett, M. S. , Hegarty, D. M. , Habecker, B. A. , & Aicher, S. A (2022). Distinct morphology of cardiac‐ and brown adipose tissue‐projecting neurons in the stellate ganglia of mice. Physiological Reports, 10(10), e15334. 35621038 10.14814/phy2.15334 PMC9136702

[eph13768-bib-0004] Beach, T. G. , Adler, C. H. , Sue, L. I. , Vedders, L. , Lue, L. , White Iii, C. L. , Akiyama, H. , Caviness, J. N. , Shill, H. A. , Sabbagh, M. N. , Walker, D. G , & Arizona Parkinson's Disease C . (2010). Multi‐organ distribution of phosphorylated α‐synuclein histopathology in subjects with Lewy body disorders. Acta Neuropathologica, 119(6), 689–702. 20306269 10.1007/s00401-010-0664-3 PMC2866090

[eph13768-bib-0005] Blackwell, D. J. , Schmeckpeper, J. , & Knollmann, B. C (2022). Animal models to study cardiac arrhythmias. Circulation Research, 130(12), 1926–1964. 35679367 10.1161/CIRCRESAHA.122.320258 PMC9202503

[eph13768-bib-0006] Borghammer, P. , Horsager, J. , Andersen, K. , Van Den Berge, N. , Raunio, A. , Murayama, S. , Parkkinen, L. , & Myllykangas, L. (2021). Neuropathological evidence of body‐first vs. brain‐first Lewy body disease. Neurobiology of Disease, 161, 105557. 34763110 10.1016/j.nbd.2021.105557

[eph13768-bib-0007] Braak, H. , Del Tredici, K. , Rub, U. , de Vos, R. A. , Jansen Steur, E. N. , & Braak, E. (2003). Staging of brain pathology related to sporadic Parkinson's disease. Neurobiology of Aging, 24(2), 197–211. 12498954 10.1016/s0197-4580(02)00065-9

[eph13768-bib-0008] Chaudhuri, K. R. , Healy, D. G. , Schapira, A. H , & National Institute for Clinical E . (2006). Non‐motor symptoms of Parkinson's disease: diagnosis and management. Lancet Neurology, 5(3), 235–245. 16488379 10.1016/S1474-4422(06)70373-8

[eph13768-bib-0009] Clyburn, C. , Andresen, M. C. , Ingram, S. L. , & Habecker, B. A (2022). Untangling peripheral sympathetic neurocircuits. Frontiers in Cardiovascular Medicine, 9, 842656. 35224065 10.3389/fcvm.2022.842656 PMC8866570

[eph13768-bib-0010] Cuthbertson, S. , LeDoux, M. S. , Jones, S. , Jones, J. , Zhou, Q. , Gong, S. , Ryan, P. , & Reiner, A. (2003). Localization of preganglionic neurons that innervate choroidal neurons of pterygopalatine ganglion. Investigative Ophthalmology & Visual Science, 44(9), 3713–3724. 12939284 10.1167/iovs.02-1207

[eph13768-bib-0011] Duffy, M. , Garland, J. , Ondruschka, B. , Paton, J. F. R. , Bardsley, E. N. , Wong, C. X. , Stables, S. , & Tse, R. (2021). Stellate ganglionitis in sudden cardiac death: a case report. Autonomic Neuroscience, 234, 102837. 34182293 10.1016/j.autneu.2021.102837

[eph13768-bib-0012] Ekmark‐Lewen, S. , Lindstrom, V. , Gumucio, A. , Ihse, E. , Behere, A. , Kahle, P. J. , Nordstrom, E. , Eriksson, M. , Erlandsson, A. , Bergstrom, J. , & Ingelsson, M. (2018). Early fine motor impairment and behavioral dysfunction in (Thy‐1)‐h[A30P] alpha‐synuclein mice. Brain and Behavior, 8(3), e00915. 29541535 10.1002/brb3.915 PMC5840441

[eph13768-bib-0013] Elliott, I. A. , DeJesus, M. , Dobaria, V. , Vaseghi, M. , Ajijola, O. A. , Shivkumar, K. , Hoftman, N. N. , Benharash, P. , Lee, J. M. , & Yanagawa, J. (2021). Minimally invasive bilateral stellate ganglionectomy for refractory ventricular tachycardia. Journal of the American College of Cardiology‐Clinical Electrophysiology, 7(4), 533–535. 10.1016/j.jacep.2020.12.001 33419708

[eph13768-bib-0014] Fleming, S. M. , Jordan, M. C. , Mulligan, C. K. , Masliah, E. , Holden, J. G. , Millard, R. W. , Chesselet, M. F. , & Roos, K. P (2013). Impaired baroreflex function in mice overexpressing alpha‐synuclein. Frontiers in Neurology, 4, 103. 23888153 10.3389/fneur.2013.00103 PMC3719027

[eph13768-bib-0015] Goncalves, V. C. , Cuenca‐Bermejo, L. , Fernandez‐Villalba, E. , Martin‐Balbuena, S. , da, S. , Fernandes, M. J. , Scorza, C. A. , & Herrero, M. T (2022). Heart matters: cardiac dysfunction and other autonomic changes in Parkinson's disease. The Neuroscientist, 28, 530–542. 33583239 10.1177/1073858421990000

[eph13768-bib-0016] Gries, M. , Christmann, A. , Schulte, S. , Weyland, M. , Rommel, S. , Martin, M. , Baller, M. , Roth, R. , Schmitteckert, S. , Unger, M. , Liu, Y. , Sommer, F. , Muhlhaus, T. , Schroda, M. , Timmermans, J. P. , Pintelon, I. , Rappold, G. A. , Britschgi, M. , Lashuel, H. , …, Schafer, K. H (2021). Parkinson mice show functional and molecular changes in the gut long before motoric disease onset. Molecular Neurodegeneration, 16(1), 34. 34078425 10.1186/s13024-021-00439-2 PMC8170976

[eph13768-bib-0017] Hedger, J. H. , & Webber, R. H (1976). Anatomical study of the cervical sympathetic trunk and ganglia in the albino rat (*Mus norvegicus albinus*). Acta Anatomica, 96(2), 206–217. 970104 10.1159/000144674

[eph13768-bib-0018] Iwanaga, K. , Wakabayashi, K. , Yoshimoto, M. , Tomita, I. , Satoh, H. , Takashima, H. , Satoh, A. , Seto, M. , Tsujihata, M. , & Takahashi, H. (1999). Lewy body‐type degeneration in cardiac plexus in Parkinson's and incidental Lewy body diseases. Neurology, 52(6), 1269–1271. 10214756 10.1212/wnl.52.6.1269

[eph13768-bib-0019] Jain, S. , & Goldstein, D. S (2012). Cardiovascular dysautonomia in Parkinson disease: from pathophysiology to pathogenesis. Neurobiology of Disease, 46(3), 572–580. 22094370 10.1016/j.nbd.2011.10.025 PMC3299874

[eph13768-bib-0020] Javanshiri, K. , Drakenberg, T. , Haglund, M. , & Englund, E. (2022). Cardiac alpha‐synuclein is present in alpha‐synucleinopathies. Journal of Parkinson's Disease, 12(4), 1125–1131. 10.3233/JPD-223161 PMC9198726 35275559

[eph13768-bib-0021] Jost, W. H (2003). Autonomic dysfunctions in idiopathic Parkinson's disease. Journal of Neurology, 250(S1), i28–i30. 12761632 10.1007/s00415-003-1105-z

[eph13768-bib-0022] Kahle, P. J. , Neumann, M. , Ozmen, L. , Muller, V. , Jacobsen, H. , Schindzielorz, A. , Okochi, M. , Leimer, U. , van Der Putten, H. , Probst, A. , Kremmer, E. , Kretzschmar, H. A. , & Haass, C. (2000). Subcellular localization of wild‐type and Parkinson's disease‐associated mutant α‐synuclein in human and transgenic mouse brain. Journal of Neuroscience, 20(17), 6365–6373. 10964942 10.1523/JNEUROSCI.20-17-06365.2000 PMC6772969

[eph13768-bib-0023] Kim, S. , Kwon, S. H. , Kam, T. I. , Panicker, N. , Karuppagounder, S. S. , Lee, S. , Lee, J. H. , Kim, W. R. , Kook, M. , Foss, C. A. , Shen, C. , Lee, H. , Kulkarni, S. , Pasricha, P. J. , Lee, G. , Pomper, M. G. , Dawson, V. L. , Dawson, T. M. , & Ko, H. S (2019). Transneuronal propagation of pathologic α‐synuclein from the gut to the brain models Parkinson's disease. Neuron, 103(4), 627–641.e7. 31255487 10.1016/j.neuron.2019.05.035 PMC6706297

[eph13768-bib-0024] Klegeris, A. , Pelech, S. , Giasson, B. I. , Maguire, J. , Zhang, H. , McGeer, E. G. , & McGeer, P. L (2008). Alpha‐synuclein activates stress signaling protein kinases in THP‐1 cells and microglia. Neurobiology of Aging, 29(5), 739–752. 17166628 10.1016/j.neurobiolaging.2006.11.013

[eph13768-bib-0025] Kollias, G. , Spanopoulou, E. , Grosveld, F. , Ritter, M. , Beech, J. , & Morris, R. (1987). Differential regulation of a Thy‐1 gene in transgenic mice. Proceedings of the National Academy of Sciences, USA, 84(6), 1492–1496. 10.1073/pnas.84.6.1492 PMC304460 2882505

[eph13768-bib-0026] Kruger, R. , Kuhn, W. , Muller, T. , Woitalla, D. , Graeber, M. , Kosel, S. , Przuntek, H. , Epplen, J. T. , Schols, L. , & Riess, O. (1998). Ala30Pro mutation in the gene encoding α synuclein in Parkinson's disease. Nature Genetics, 18(2), 106–108. 9462735 10.1038/ng0298-106

[eph13768-bib-0027] Lee, B. , Edling, C. , Ahmad, S. , LeBeau, F. E. N. , Tse, G. , & Jeevaratnam, K. (2023). Clinical and non‐clinical cardiovascular disease associated pathologies in Parkinson's disease. International Journal of Molecular Sciences, 24, 12601. 37628780 10.3390/ijms241612601 PMC10454288

[eph13768-bib-0028] Lee, Y. S. , Wie, C. , Pew, S. , & Kling, J. M (2022). Stellate ganglion block as a treatment for vasomotor symptoms: clinical application. Cleveland Clinic Journal of Medicine, 89(3), 147–153. 35232827 10.3949/ccjm.89a.21032

[eph13768-bib-0029] Li, J. , Uversky, V. N. , & Fink, A. L (2001). Effect of familial Parkinson's disease point mutations A30P and A53T on the structural properties, aggregation, and fibrillation of human α‐synuclein. Biochemistry, 40(38), 11604–11613. 11560511 10.1021/bi010616g

[eph13768-bib-0030] Li, Y. , Wang, J. , Li, X. , Jing, W. , Omorodion, I. , & Liu, L. (2021). Association between heart rate variability and Parkinson's disease: a meta‐analysis. Current Pharmaceutical Design, 27(17), 2056–2067. 32888281 10.2174/1871527319666200905122222

[eph13768-bib-0031] Mahul‐Mellier, A. L. , Burtscher, J. , Maharjan, N. , Weerens, L. , Croisier, M. , Kuttler, F. , Leleu, M. , Knott, G. W. , & Lashuel, H. A (2020). The process of Lewy body formation, rather than simply α‐synuclein fibrillization, is one of the major drivers of neurodegeneration. Proceedings of the National Academy of Sciences, USA, 117(9), 4971–4982. 10.1073/pnas.1913904117 PMC7060668 32075919

[eph13768-bib-0032] Mehra, S. , Sahay, S. , & Maji, S. K (2019). α‐Synuclein misfolding and aggregation: Implications in Parkinson's disease pathogenesis. Biochimica et Biophysica Acta. Proteins and Proteomics, 1867(10), 890–908. 30853581 10.1016/j.bbapap.2019.03.001

[eph13768-bib-0033] Miki, Y. , Mori, F. , Wakabayashi, K. , Kuroda, N. , & Orimo, S. (2009). Incidental Lewy body disease restricted to the heart and stellate ganglia. Movement Disorders, 24(15), 2299–2301. 19795472 10.1002/mds.22775

[eph13768-bib-0034] Narhi, L. , Wood, S. J. , Steavenson, S. , Jiang, Y. , Wu, G. M. , Anafi, D. , Kaufman, S. A. , Martin, F. , Sitney, K. , Denis, P. , Louis, J. C. , Wypych, J. , Biere, A. L. , & Citron, M. (1999). Both familial Parkinson's disease mutations accelerate α‐synuclein aggregation. Journal of Biological Chemistry, 274(14), 9843–9846. 10092675 10.1074/jbc.274.14.9843

[eph13768-bib-0035] Navarro‐Otano, J. , Gelpi, E. , Mestres, C. A. , Quintana, E. , Rauek, S. , Ribalta, T. , Santiago, V. , & Tolosa, E. (2013). Alpha‐synuclein aggregates in epicardial fat tissue in living subjects without parkinsonism. Parkinsonism & Related Disorders, 19(1), 27–31. discussion 27. 22858179 10.1016/j.parkreldis.2012.07.005

[eph13768-bib-0036] Navickaite, I. , Pauziene, N. , & Pauza, D. H (2021). Anatomical evidence of non‐parasympathetic cardiac nitrergic nerve fibres in rat. Journal of Anatomy, 238(1), 20–35. 32790077 10.1111/joa.13291 PMC7755078

[eph13768-bib-0037] Nishida, N. , Yoshida, K. , & Hata, Y. (2017). Sudden unexpected death in early Parkinson's disease: neurogenic or cardiac death? Cardiovascular Pathology, 30, 19–22. 28666147 10.1016/j.carpath.2017.06.001

[eph13768-bib-0038] Pankratz, N. , Nichols, W. C. , Elsaesser, V. E. , Pauciulo, M. W. , Marek, D. K. , Halter, C. A. , Wojcieszek, J. , Rudolph, A. , Pfeiffer, R. F. , Foroud, T. , & Parkinson Study Group PI . (2009). Alpha‐synuclein and familial Parkinson's disease. Movement Disorders, 24(8), 1125–1131. 19412953 10.1002/mds.22524 PMC3397145

[eph13768-bib-0039] Rajendran, P. S. , Challis, R. C. , Fowlkes, C. C. , Hanna, P. , Tompkins, J. D. , Jordan, M. C. , Hiyari, S. , Gabris‐Weber, B. A. , Greenbaum, A. , Chan, K. Y. , Deverman, B. E. , Munzberg, H. , Ardell, J. L. , Salama, G. , Gradinaru, V. , & Shivkumar, K. (2019). Identification of peripheral neural circuits that regulate heart rate using optogenetic and viral vector strategies. Nature Communications, 10(1), 1944. 10.1038/s41467-019-09770-1 PMC6486614 31028266

[eph13768-bib-0040] Rizzo, S. , Basso, C. , Troost, D. , Aronica, E. , Frigo, A. C. , Driessen, A. H. , Thiene, G. , Wilde, A. A. , & van der Wal, A. C (2014). T‐cell‐mediated inflammatory activity in the stellate ganglia of patients with ion‐channel disease and severe ventricular arrhythmias. Circulation: Arrhythmia and Electrophysiology, 7(2), 224–229. 24532560 10.1161/CIRCEP.113.001184

[eph13768-bib-0041] Schapira, A. H. V. , Chaudhuri, K. R. , & Jenner, P. (2017). Non‐motor features of Parkinson disease. Nature Reviews Neuroscience, 18(7), 435–450. 28592904 10.1038/nrn.2017.62

[eph13768-bib-0042] Scherschel, K. , Brauninger, H. , Glufke, K. , Jungen, C. , Klocker, N. , & Meyer, C. (2020). Location, dissection, and analysis of the murine stellate ganglion. Journal of Visualized Experiments, (166), e62026. 10.3791/62026 33427236

[eph13768-bib-0043] Scorza, F. A. , Fiorini, A. C. , Scorza, C. A. , & Finsterer, J. (2018). Cardiac abnormalities in Parkinson's disease and Parkinsonism. Journal of Clinical Neuroscience, 53, 1–5. 29706419 10.1016/j.jocn.2018.04.031

[eph13768-bib-0044] Sharma, S. , Littman, R. , Tompkins, J. D. , Arneson, D. , Contreras, J. , Dajani, A. H. , Ang, K. , Tsanhani, A. , Sun, X. , Jay, P. Y. , Herzog, H. , Yang, X. , & Ajijola, O. A (2023). Tiered sympathetic control of cardiac function revealed by viral tracing and single cell transcriptome profiling. eLife, 12, e86295. 37162194 10.7554/eLife.86295 PMC10212561

[eph13768-bib-0045] Sumikura, H. , Takao, M. , Hatsuta, H. , Ito, S. , Nakano, Y. , Uchino, A. , Nogami, A. , Saito, Y. , Mochizuki, H. , & Murayama, S. (2015). Distribution of α‐synuclein in the spinal cord and dorsal root ganglia in an autopsy cohort of elderly persons. Acta Neuropathologica Communications, 3(1), 57. 26374630 10.1186/s40478-015-0236-9 PMC4571135

[eph13768-bib-0046] Takahashi, M. , Ikemura, M. , Oka, T. , Uchihara, T. , Wakabayashi, K. , Kakita, A. , Takahashi, H. , Yoshida, M. , Toru, S. , Kobayashi, T. , & Orimo, S. (2015). Quantitative correlation between cardiac MIBG uptake and remaining axons in the cardiac sympathetic nerve in Lewy body disease. Journal of Neurology, Neurosurgery, and Psychiatry, 86(9), 939–944. 25935891 10.1136/jnnp-2015-310686

[eph13768-bib-0047] Vidal, M. , Morris, R. , Grosveld, F. , & Spanopoulou, E. (1990). Tissue‐specific control elements of the Thy‐1 gene. EMBO Journal, 9(3), 833–840. 1968831 10.1002/j.1460-2075.1990.tb08180.x PMC551743

[eph13768-bib-0048] Virdi, G. S. , Choi, M. L. , Evans, J. R. , Yao, Z. , Athauda, D. , Strohbuecker, S. , Nirujogi, R. S. , Wernick, A. I. , Pelegrina‐Hidalgo, N. , Leighton, C. , Saleeb, R. S. , Kopach, O. , Alrashidi, H. , Melandri, D. , Perez‐Lloret, J. , Angelova, P. R. , Sylantyev, S. , Eaton, S. , Heales, S. , …, Gandhi, S. (2022). Protein aggregation and calcium dysregulation are hallmarks of familial Parkinson's disease in midbrain dopaminergic neurons. NPJ Parkinson's Disease, 8(1), 162. 10.1038/s41531-022-00423-7 PMC9691718 36424392

[eph13768-bib-0049] Wakabayashi, K. , Tanji, K. , Odagiri, S. , Miki, Y. , Mori, F. , & Takahashi, H. (2013). The Lewy body in Parkinson's disease and related neurodegenerative disorders. Molecular Neurobiology, 47(2), 495–508. 22622968 10.1007/s12035-012-8280-y

[eph13768-bib-0050] Wang, X. J. , Ma, M. M. , Zhou, L. B. , Jiang, X. Y. , Hao, M. M. , Teng, R. K. F. , Wu, E. , Tang, B. S. , Li, J. Y. , Teng, J. F. , & Ding, X. B (2020). Autonomic ganglionic injection of α‐synuclein fibrils as a model of pure autonomic failure α‐synucleinopathy. Nature Communications, 11(1), 934. 10.1038/s41467-019-14189-9 PMC7028908 32071315

