# Supplementary material for: Intact microdissection of stellate ganglia in a Parkinson's disease model reveals aggregation of mutant human α‐synuclein in their cell bodies
Source: Exp Physiol. 2025 Feb 21;110(6):866–76. doi: 10.1113/EP092261 (PMC12128452; doi:10.1113/EP092261)
Supplement: Supplementary file 1 — Supplementary Figure 1. Immunolabelling of sympathetic and parasympathetic markers in murine stellate ganglia. Supplementary Figure 2. Investigation of TH and hαSYN overlapping area in stellate ganglia in [A30PαSYN] mice. Supplementary Figure 3. The mutant protein distribution in the cardiopulmonary pole of stellate ganglia. [file EPH-110-866-s002.docx]

Supporting information for “Intact Microdissection of Stellate Ganglia in Parkinson's Disease Model Reveal Aggregation of Mutant Human-Alpha Synuclein in their Cell Bodies.”

Bonn Lee^1^, Shiraz Ahmad^1^, Charlotte E. Edling^1^, Fiona E.N. LeBeau^2^, Kamalan Jeevaratnam^1^

1. School of Veterinary Medicine, Faculty of Health and Medical Science, University of Surrey, UK; VSM Building, Daphne Jackson Road, Guildford, GU2 7AL.

2. Biosciences Institute, Faculty of Medical Sciences, University of Newcastle, UK; The Medical School, Framlington Place, Newcastle upon Tyne, NE2 4HH.

*Corresponding author


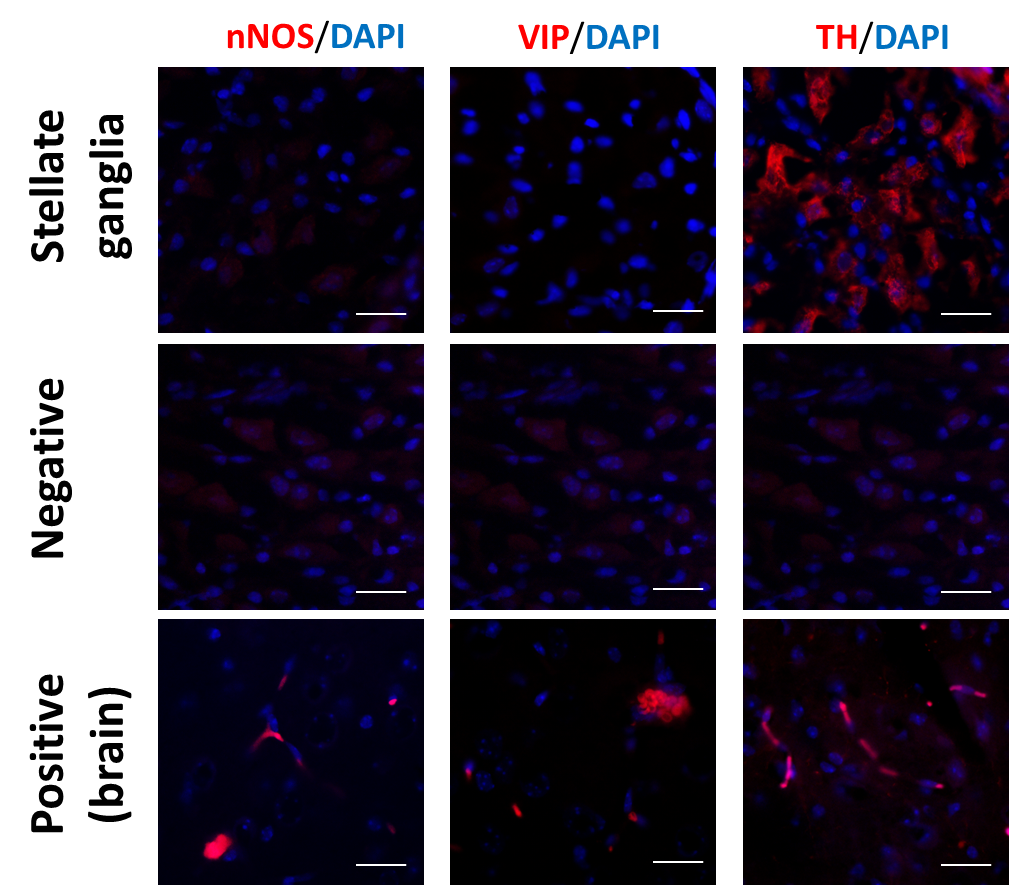


**Supplementary Figure 1.** Immunolabelling of sympathetic and parasympathetic markers in murine stellate ganglia. Representative images of sympathetic marker TH, and parasympathetic markers nNOS and VIP expression in the stellate ganglia. Parasympathetic markers were not detected in stellate ganglia, while the sympathetic marker was detected in the neuronal cell bodies, Scale bar 20μm.


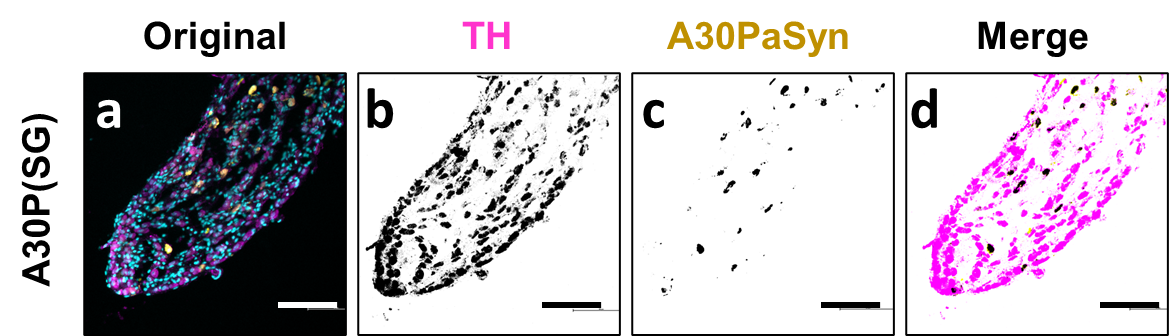


**Supplementary Figure 2.** Investigation of TH and hαSYN overlapping area in stellate ganglia in [A30PαSYN] mice. The image from Figure 4 were converted to binary color images to enable us to distinguish minute expression, and we could confirm that [A30P]αSYN protein was expressed solely in the neuronal cell bodies. (a) original image, (b) area of anti-TH positive; (c) area of anti-hαSYN positive (d) overlapped area between anti-TH and anti-hαSYN. Scale bar 100μm.


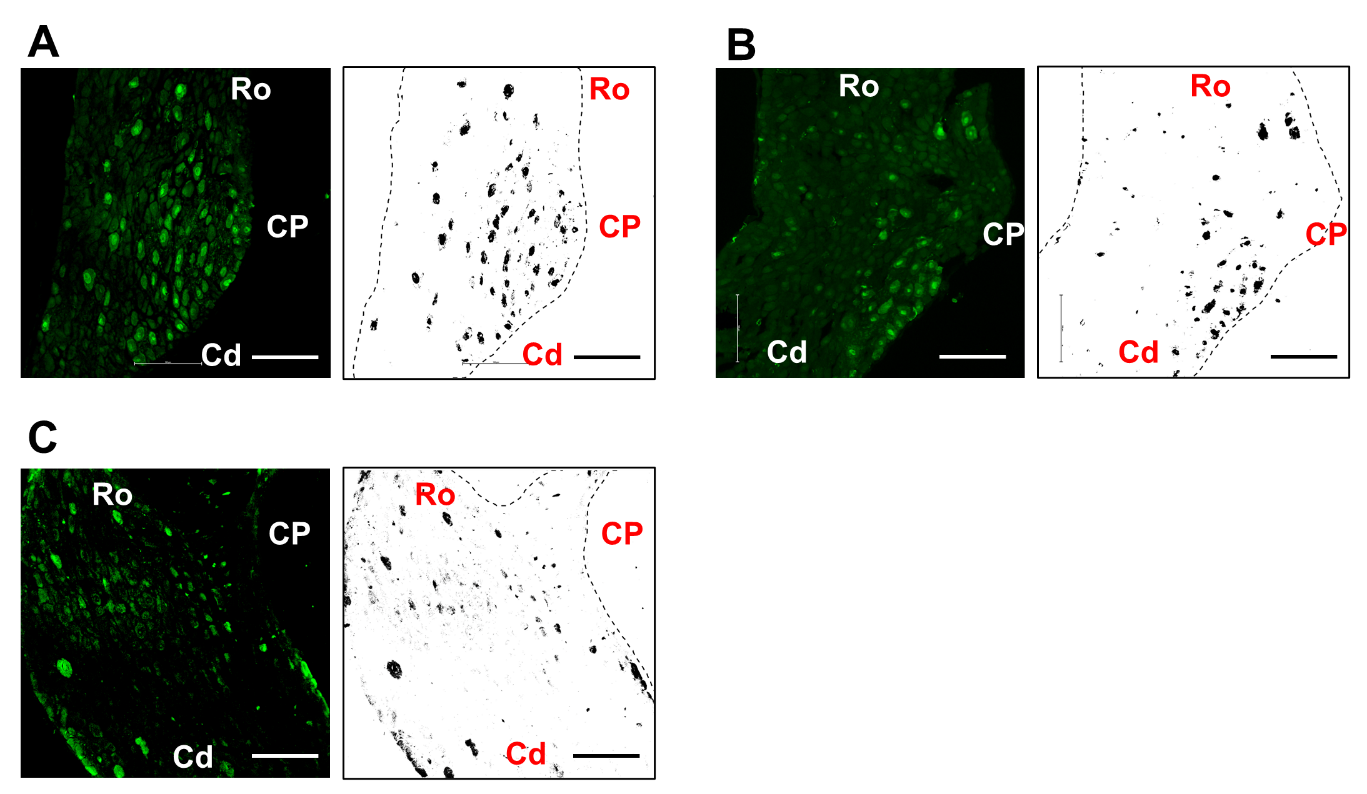


**Supplementary Figure 3.** The mutant protein distribution in the cardiopulmonary pole of stellate ganglia. The anti-A30PαSYN was located at the stellate ganglia (left), then the images were converted to binary images (right). The images showing cardiopulmonary pole were presented. Ro, rostral orientation, Cd, caudal orientation, CP, cardiopulmonary pole. Scale bar, 100μm.
